# UV induced changes in proteome of rats plasma are reversed by dermally applied cannabidiol

**DOI:** 10.1038/s41598-021-00134-8

**Published:** 2021-10-19

**Authors:** Agnieszka Gęgotek, Sinemyiz Atalay, Elżbieta Skrzydlewska

**Affiliations:** Department of Analytical Chemistry, Medical University of Bialystok, Mickiewicza 2D, 15-222 Bialystok, Poland

**Keywords:** Biochemistry, Molecular biology, Molecular medicine

## Abstract

UV radiation is known to induce a multiple changes in the metabolism of skin-building cells, what can affect the functioning not only neighboring cells, but also, following signal transduction releasing into the blood vessels, the entire body. Therefore, the aim of this study was to analyze the proteomic disturbances occurred in plasma of chronically UVA/UVB irradiated rats and define the effect on these changes of skin topically applied cannabidiol (CBD). Obtained results showed significant changes in the expression of numerous anti-inflammatory and signaling proteins including: NFκB inhibitor, 14-3-3 protein, protein kinase C, keratin, and protein S100 after UV irradiation and CBD treatment. Moreover, the effects of UVA and UVB were manifested by increased level of lipid peroxidation products—protein adducts formation. CBD partially prevented all of these changes, but in a various degree depending on the UV radiation type. Moreover, topical treatment with CBD resulted in the penetration of CBD into the blood and, as a consequence, in direct modifications to the plasma protein structure by creating CBD adducts with molecules, such as proline-rich protein 30, transcription factor 19, or N-acetylglucosamine-6-sulfatase, what significantly changed the activity of these proteins. In conclusion, it may be suggested that CBD applied topically may be an effective compound against systemic UV-induced oxidative stress, but its effectiveness requires careful analysis of CBD's effects on other tissues of the living organism.

## Introduction

It is known that UV radiation, as the main environmental physical factor that reaches human skin, causes many changes in the metabolism of skin-building cells^1^. Depending on the type of UV radiation (UVA or UVB), different types of cells, at different levels, are exposed to this factor to a different extent. UVA, which reaches the epidermis, as well as the dermis, including its basic cells—fibroblasts, is known mainly as an activator of photosensitive compounds such as chromophores^2^. On the other hand, high-energy UVB is largely retained in the epidermis by keratinocytes and directly in these cells induces changes at the molecular level. Therefore, both types of radiation stimulates skin cells to the generation of signals to produce rapid (neural) or slow (humoral or immune) responses at the local and systemic levels mediated by the skin neuroendocrine system^3,4^. It is well known that skin, as one of the largest organs of the human body, both receives information about the surrounding environment and through expression of signaling molecules, stimulates action of a well-developed neuroendocrine system, resulting in maintenance of body’s homeostasis^5^. This natural platform of signal exchange between environment and internal organs is subjected to neurohormonal regulation, but also to produce neuropeptides, biogenic amines, melatonin, opioids, acetylcholine, steroids, secosteroids, growth factors, cytokines, as well as endocannabinoids^4,6,7^. It has been shown that skin UV irradiation leads to the enhanced β-endorphin, corticosterone, corticotropin-releasing and adrenocorticotropic hormone production accompanied by rapid immunosuppressive effects with decreased body’s immunity^8,9^. Moreover, skin exposure to UVA or UVB radiation results in disturbances in the generation and removal of reactive oxygen species, leading to oxidative stress, which results in oxidative modifications of nucleic acids, proteins and lipids^10^. As a consequence, dysfunctional molecules are formed that hinder the efficient metabolism of cells and the normal functioning of the skin. On the other hand, these processes also generate signaling molecules, including pro-inflammatory/pro-apoptotic proteins, as well as lipid peroxidation products such as reactive aldehydes and isoprostanes^11^. All these molecules can be released into the blood vessels and cause a pro-inflammatory reaction not only in neighboring cells, but also in the entire body^3^. So far, with the exception of neuroendocrine system activity, the harmful oxidative effects of UV radiation on body tissues have been proven mainly in the liver of UV irradiated mice, where a significant increase in triglycerides was accompanied by a decrease in the level of glutathione (GSH) and the activity of GSSG reductase, superoxide dismutase and catalase^12,13^. Moreover, UV irradiated mice have elevated level of blood urocanic acid and glutamate, which transferred into brain significantly affects synapses^14^. So far, systemic human studies showed only the effect of UV radiation on the blood pressure that is lowered by UV radiation through the skin's increased generation of nitric oxide^15,16^.

With regard to the above harmful properties of UV radiation in this study the cannabidiol (CBD) is proposed as a compound that can effectively counteract the effects of UV radiation. CBD is a non-psychoactive phytocannabinoid mainly obtained from *Cannabis sativa* L. leaves. Its action is based primarily on the interaction with the endocannabinoid system, consisting of endocannabinoids, as well as their specific transmembrane receptors^17^. CBD inhibits endocannabinoid pro-inflammatory signaling directly by negative modulation of CB1 activity^18^, or indirectly by interaction with the main agonist of this receptor—anandamide^19^. CBD antioxidant and anti-inflammatory properties leading to enhanced antioxidant enzymes activity, as well as silencing the TNFα/NFκB pathway^19,20^ are also visible in the context of UV irradiated skin fibroblasts and keratinocytes^21–23^, therefore, it is more and more frequently used especially in the skincare products^24^. So far, the positive effects of CBD were also used in the design of anti-cancer therapies^25^, however, it has also been shown to have anticonvulsive, antianxiety, antinausea, and antirheumatoid properties^26^.

According to the such a broad spectrum of CBD action, connected with its properties of penetration through biological membranes and poly-layer cells cultures^21,22^, as well as its possibility of infiltration from the skin into the blood^27^, it is suggested that CBD may have significant impact on the oxidative, as well as pro-inflammatory changes induces by UV radiation. Therefore, the aim of this study was to analyze the changes in the proteomic profile of plasma from chronically UVA or UVB irradiated rats and define the effect on these changes of skin topically applied CBD.

## Materials and methods

All the methods were performed in accordance with the ARRIVE guidelines.

### Animals treatment

The experiment was carried out on nude rats (RH-FOXN1RNU, males weighing 260–302 g) aged for 8–9 weeks. The rats were kept under standard conditions (12 h (h) light/12 h dark cycles) and fed a standard pellet diet, containing a mix of various dietary constituents, including proteins, fibres, and minerals^28^. All the procedures and experimental protocols on animals were approved by the Local Ethics Committee for Animal Experiments in Olsztyn, Poland (resolution No. 37/2019 of 26.04.2019). The rats were divided into six groups of six rats each:

1. [CTR]: control rats, for 4 weeks every 12 h, treated topically by the 20 min application of non-toxic hydrophilic petrolatum to the animal's back, which was then removed.
2. [CBD]: rats, which back 4 weeks every 12 h was treated topically for 20 min with 2.5 g CBD in 100 g petrolatum^29^.
3. [UVA]: rat’s back every 48 h for 4 weeks was exposed to UVA radiation in increasing doses from 0.5 to 5 J/cm^2^^30^. Doses were almost doubled every 3 exposures, what gives in total 27.25 J/cm^2^ applied UVA radiation.
4. [UVA + CBD]: topically treated for 20 min with 2.5 g CBD in 100 g petrolatum, which was applied to the back of the animal every 12 h for 4 weeks and then it was removed. Every 48 h, before CBD application, the skin of the back was exposed to the UVA radiation in increasing doses from 0.5 to 5 J/cm^2^ every 48 h for 4 weeks, as described for [UVA] group.
5. [UVB]: rat’s back every 48 h for 4 weeks was exposed to UVB radiation in increasing doses from 0.02 to 2 J/cm^2^^31^. Doses were almost doubled every 2 exposures, what gives in total 6 J/cm^2^ applied UVB radiation.
6. [UVB + CBD]: topically treated for 20 min with 2.5 g CBD in 100 g petrolatum, which was applied to the back of the animal every 12 h for 4 weeks and then it was removed. Every 48 h, before CBD application, the skin of the back was exposed to the UVB radiation in increasing doses from 0.02 to 2 J/cm^2^ every 48 h for 4 weeks, as described for [UVB] group.

The skin on rat’s back was irradiated using lamp (Cosmedico, Stuttgart, Germany) with UVA (max. emission peak at 368 nm) or UVB (max. emission peak at 311 nm) emitters (PL-S 9 W; Philips, Amsterdam, Netherlands) applied in the dermatology for human skin diseases treatment. The spectrum of UV emitters used in the experiments are showed in Supplementary figure S1. The radiation doses used in experiment were selected according to the standard protocols used in the phototherapy of human skin diseases, including psoriasis. To ensure the constant distance (~ 2 cm) between the irradiated skin and lamp the plastic combs were used, what allows to maintain a required dose of radiation over a time and protects against skin overheating.

Due to the potential protective effect of petrolatum against skin drying and pro-inflammatory reactions^32–34^, to minimize the influence of external factors on the metabolic changes in animal skin caused by CBD, all irradiated rats were treated topically with petroleum jelly (with or without CBD) after UV irradiation. For 20 min, which was then removed.

After a 4-week experiment, the animals were anaesthetized using inhaled isoflurane and sacrificed by cardiac excision. During this procedures, the blood from hart were taken into ethylenediaminetetraacetic acid (EDTA) tubes and centrifuged at 3000 g (4 °C) to obtain the plasma for proteomic analysis. All the methods were performed in accordance with the relevant guidelines and regulations.

### Samples preparation, electrophoretic separation and protein digestion

**Figure 1:**
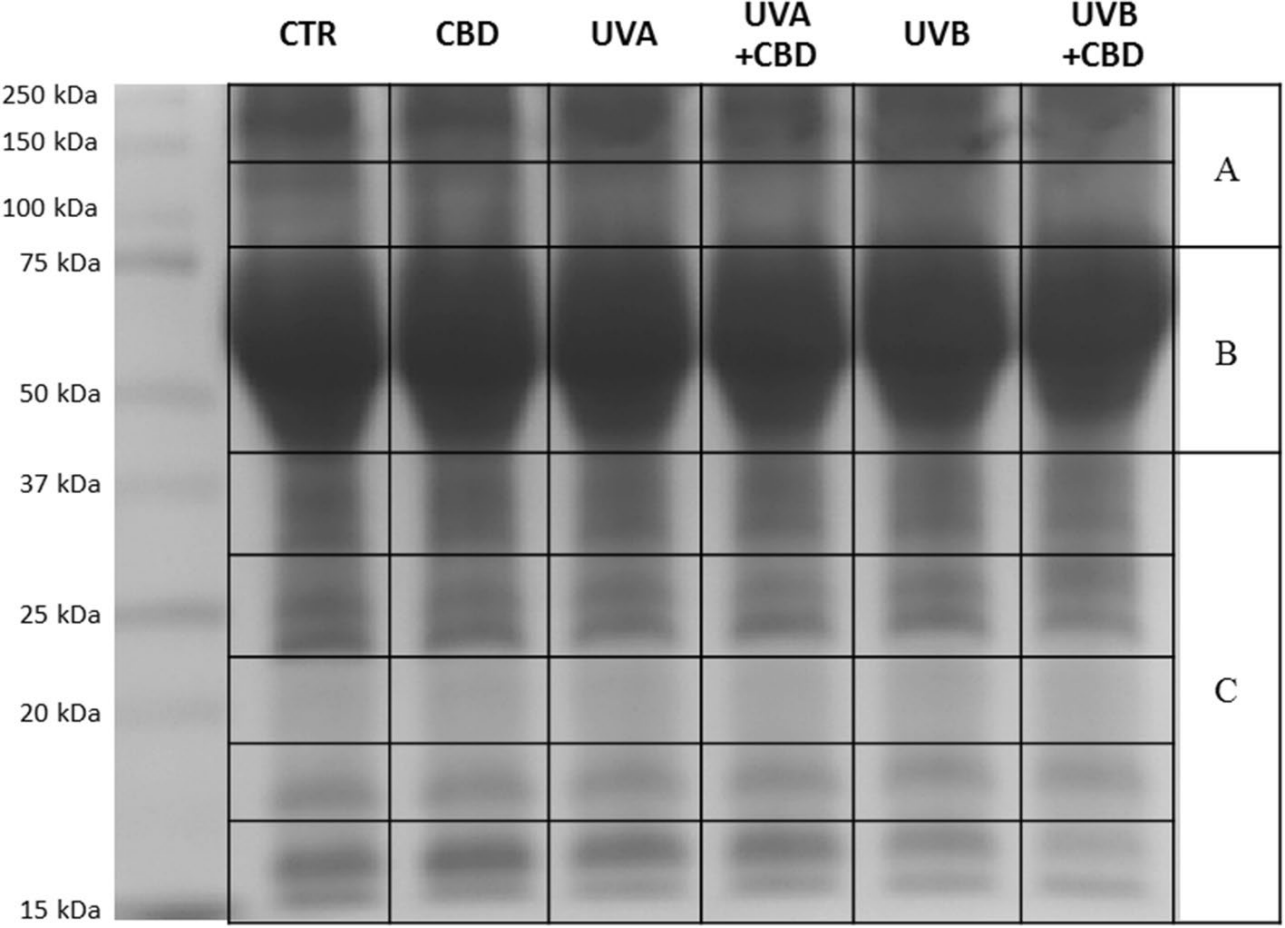
SDS-PAGE separation and staining with Coomassie Brilliant Blue R-250 of plasma proteins from the control rats (CTR) and animals topically treated with cannabidiol (2.5 g CBD in 100 g petrolatum) and/or irradiated with UVA (increasing doses from 0.5 to 5 J/cm^2^) or UVB (increasing doses from 0.02 to 2 J/cm^2^). (**A**–**C**) Protein pooling sections for proteomic analyzes.

The total protein concentration of the plasma samples was measured by Bradford assay^35^. The volume of plasma containing 30 µg of protein were mixed 1:1 (v:v) with a sample denaturing buffer (Laemmli buffer containing 5% 2-mercaptoethanol) and boiled for 7 min. Next samples were separated using 10% Tris–Glycine SDS-PAGE gels. The gels with separated proteins were fixed in methanol: acetic acid: water (4:1:5; v:v) for 1 h and stained overnight with Coomassie Brilliant Blue R-250. Following washing off, detected bands were cut from the gels, sliced and pulled into 3 sections (Fig. 1, Supplementary figure S2). The proteins in each section were reduced with 10 mM 1,4-dithiothreitol, alkylated by incubation with 50 mM iodoacetamide, and in-gel digested overnight with trypsin (Promega, Madison, WI, USA). The isolated peptide mixture was extracted from the gel, dried, and dissolved in 5% acetonitrile with 0.1% formic acid.

### Proteomic analysis

The final peptide mixture was separated using high-performance liquid chromatography system (Ultimate 3000; Dionex, Idstein, Germany) on a 150 mm × 0.075 mm PepMap RSLC capillary analytical C18 column with 2 µm particle size at a flow rate of 0.300 µL/min. The solvents gradient started at 3 min and increased to 60% eluent B (90% acetonitrile + 0.03% formic acid) for 40 min. Eluent A contained 5% acetonitrile with 0.1% formic acid. Eluated peptides were analysed using a Q Exactive HF mass spectrometer with an electrospray ionization source (ESI) (Thermo Fisher Scientific, Bremen, Germany). The conditions of the analysis by liquid chromatography-tandem mass spectrometry (LC–MS/MS) for peptide identification have been described in detail previously^36^.

### Protein identification and label-free quantification

The data were analyzed using Proteome Discoverer 2.0 (Thermo Fisher Scientific, Seattle, WA, USA). Protein label-free quantification was performed according to the signal intensities of the precursor ions. For the identification of proteins, the following search parameters were used; peptide mass tolerance set to 10 ppm, MS/MS mass tolerance set to 0.02 Da, up to two missed cleavages allowed, cysteine carbamidomethylation and carboxymethylation, methionine oxidation, as well as 4-HNE—cysteine/lysine/histidine, MDA—lysine, CBD—cysteine set as a dynamic modifications^37,38^. The levels of 4-HNE/MDA/CBD-protein adducts were estimated based on the peak intensity of peptides modified by 4-HNE. Input data were searched against the UniProtKB-SwissProt database (taxonomy: Rattus norvegicus, release 2021–02).

### Statistical analysis

Samples from each experimental group were analyzed in six independent replicates. The results from individual protein label-free quantification were log-transformed and auto-scaled (mean-centered and divided by the standard deviation of each variable) using open-source software MetaboAnalyst 5.0 (http://www.metaboanalyst.ca)^39^. The same software was used for biostatistical analysis, including univariate analysis (one-way ANOVA, Fisher’s LSD) and principal component analysis (PCA), heat maps with dendrogram creation and clustering.

### Ethics approval

All the procedures and experimental protocols on animals were approved by the Local Ethics Committee for Animal Experiments in Olsztyn, Poland (resolution No. 37/2019 of 26.04.2019).

### Consent to participate

Not applicable.

### Consent for publication

Not applicable.

## Results

**Figure 2:**
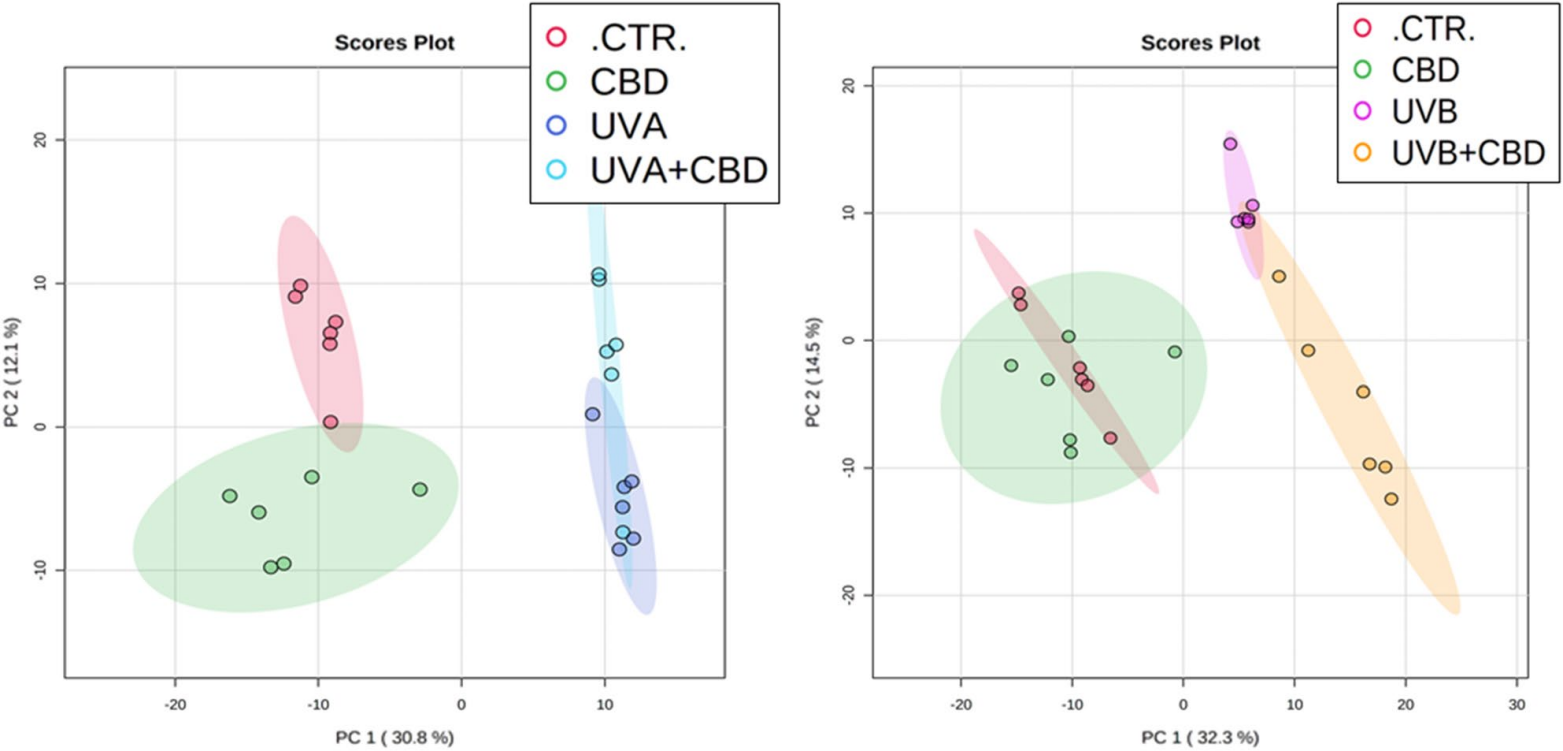
Principal component analysis (PCA) of plasma proteins from the control rats (CTR) and animals topically treated with cannabidiol (2.5 g CBD in 100 g petrolatum) and/or irradiated with UVA (increasing doses from 0.5 to 5 J/cm^2^) (**A**) or UVB (increasing doses from 0.02 to 2 J/cm^2^) (**B**). ssStatistical analysis were performed using open-source software MetaboAnalyst 5.0 (www.metaboanalyst.ca).

The conducted experiment was aimed at demonstrating the effect of topical CBD treatment on the UV-induced changes in the profiles of plasma of the rats following chronic UVA/UVB irradiation. Despite of the fact that preliminary in gel separation of proteins showed no significant differences in the band profile between examined samples (Fig. 1), the proteomic analysis allowed to determined proteins which expression or structure was affected by UV radiation and protected by CBD. Obtained results indicated 407 proteins detected and label free quantified. The expression of 318 of these proteins showed statistically significant differences between samples (267 proteins in the case of UVA/CBD vs. CTR and 295 proteins in the case of UVB/CBD treatment vs. CTR) (Supplementary table S1). The principal component analysis showed clear differentiation between UVA or UVB irradiated rats and CTR group (Fig. 2A,B). Moreover, the treatment rats with CBD did not significantly affect differentiation between CTR and CBD group, however, in the case of UV irradiated rats CBD meaningly moved the UV + CBD group away from only UVA or UVB irradiated animals (Fig. 2A,B).

**Figure 3:**
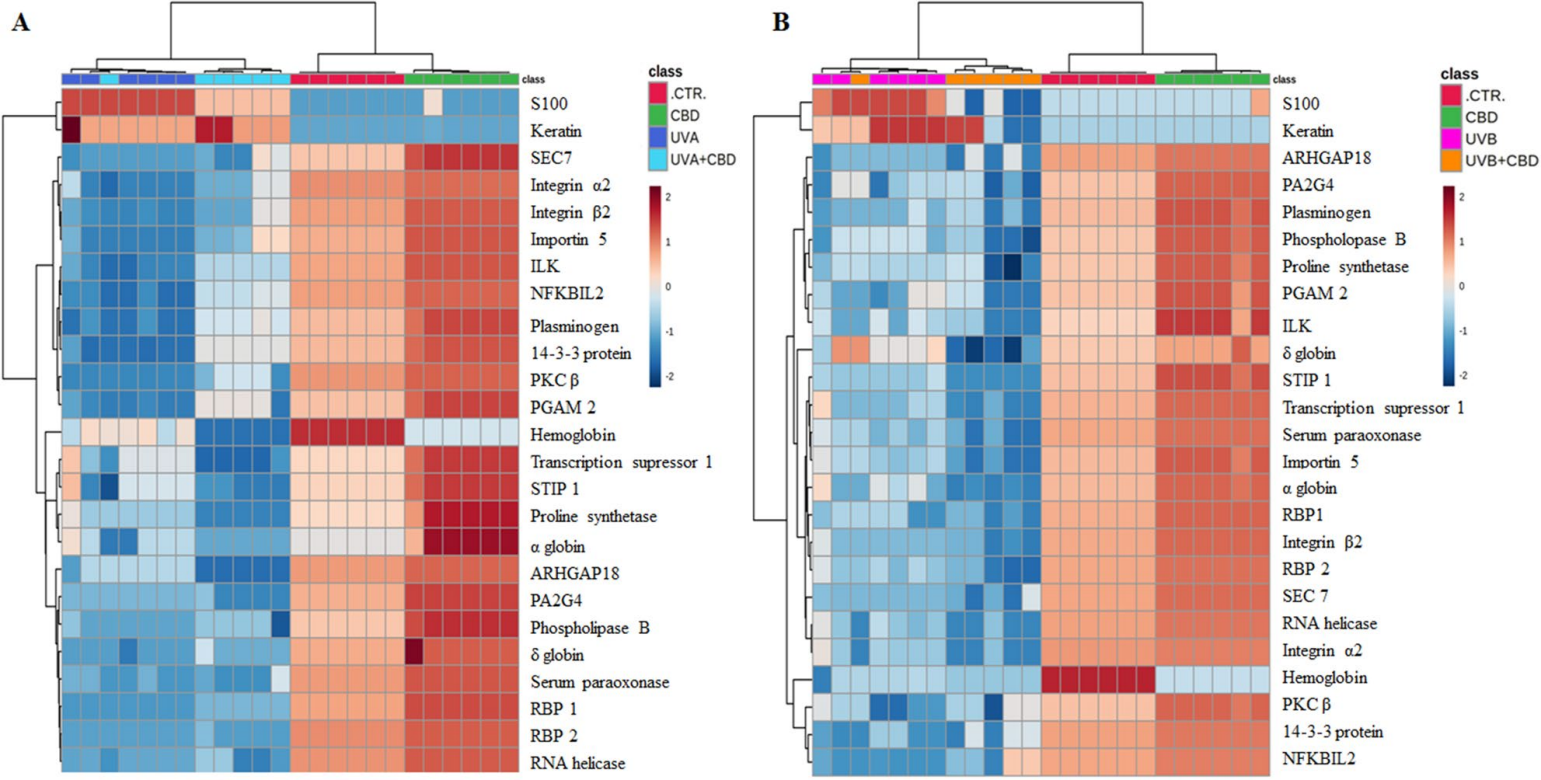
Dendrograms and heatmap for 25 top-modified ANOVA-selected proteins from plasma of control rats (CTR) and animals topically treated with cannabidiol (2.5 g CBD in 100 g petrolatum) and/or irradiated with UVA (increasing doses from 0.5 to 5 J/cm^2^) (**A**) or UVB (increasing doses from 0.02 to 2 J/cm^2^) (**B**). Statistical analysis were performed using open-source software MetaboAnalyst 5.0 (www.metaboanalyst.ca). Abbreviations, ARHGAP18, Rho GTPase activating protein 18; ILK, integrin-linked kinase; NFKBIL2, NFκB inhibitor; PA2G4, proliferation-associated 2G4; PGAM 2, phosphoglycerate mutase 2; PKC, protein kinase C; RBP, RNA-binding protein; SEC 7, transport protein 7; STIP 1, septin and tuftelin interacting protein.

Similar results were observed in the case of dendrograms, where CTR and CBD groups were clustered separately from UV treated groups, while CBD treatment following UV radiation brought the obtained profiles closer to the CTR group (Fig. 3A,B). Also the heatmaps for top 25 proteins most statistically changed according to the ANOVA allowed for visualization the strongest changes induced by chronic UVA or UVB irradiation and topical CBD treatment in the plasma proteom of tested rats (Fig. 3A,B). To the proteins whose expression has been strongly induced by UVA and UVB radiation belonged keratin and protein S100, and CBD partially prevented these changes especially in the case of UVB irradiated rats. Simultaneously, UVA and UVB radiation in a similar level decreased the expression of other mentioned top proteins, including these involved in blood coagulation (plasminogen, hemoglobin, α/δ globin and GTPase-activating protein 18 (ARHGAP18)), integrin signaling pathway (integrins α2/β2, transcription suppressor 1 and integrin-linked protein kinase (ILK)), as well as anti-inflammatory signaling (nuclear factor NFκB inhibitor, phosphoglycerate mutase 2 (PGAM2), protein kinase C (PKC), 14-3-3 protein, phospholipase B, paraoxonase). Moreover, UVA and UVB radiation directly influenced the cells growth regulation by the decreasing level of proteins involved in RNA processing (RNA helicase and proliferation-associated protein 2G4 (PA2G4)), transporting (protein Sec7, retinol-binding protein (RBP1/2) and importin) and protein conformation (stress-induced-phosphoprotein 1 (STIP1) and proline synthetase). CBD treatment following UVA or UVB radiation caused quantitative changes through their additional stimulation or strong protection against them. CBD treatment following UVA significantly prevented UVA-induced reduction in the level of proteins involved in anti-inflammatory and integrin-dependent signaling pathways and additionally decreased expression of the proteins responsible for blood coagulation, RNA processing and protein conformation. At the same time, the effects of CBD on the UVB-treated group were more diverse, but overall, most of these proteins were further reduced by CBD in UVB-irradiated animals (Fig. 3A,B).

**Figure 4:**
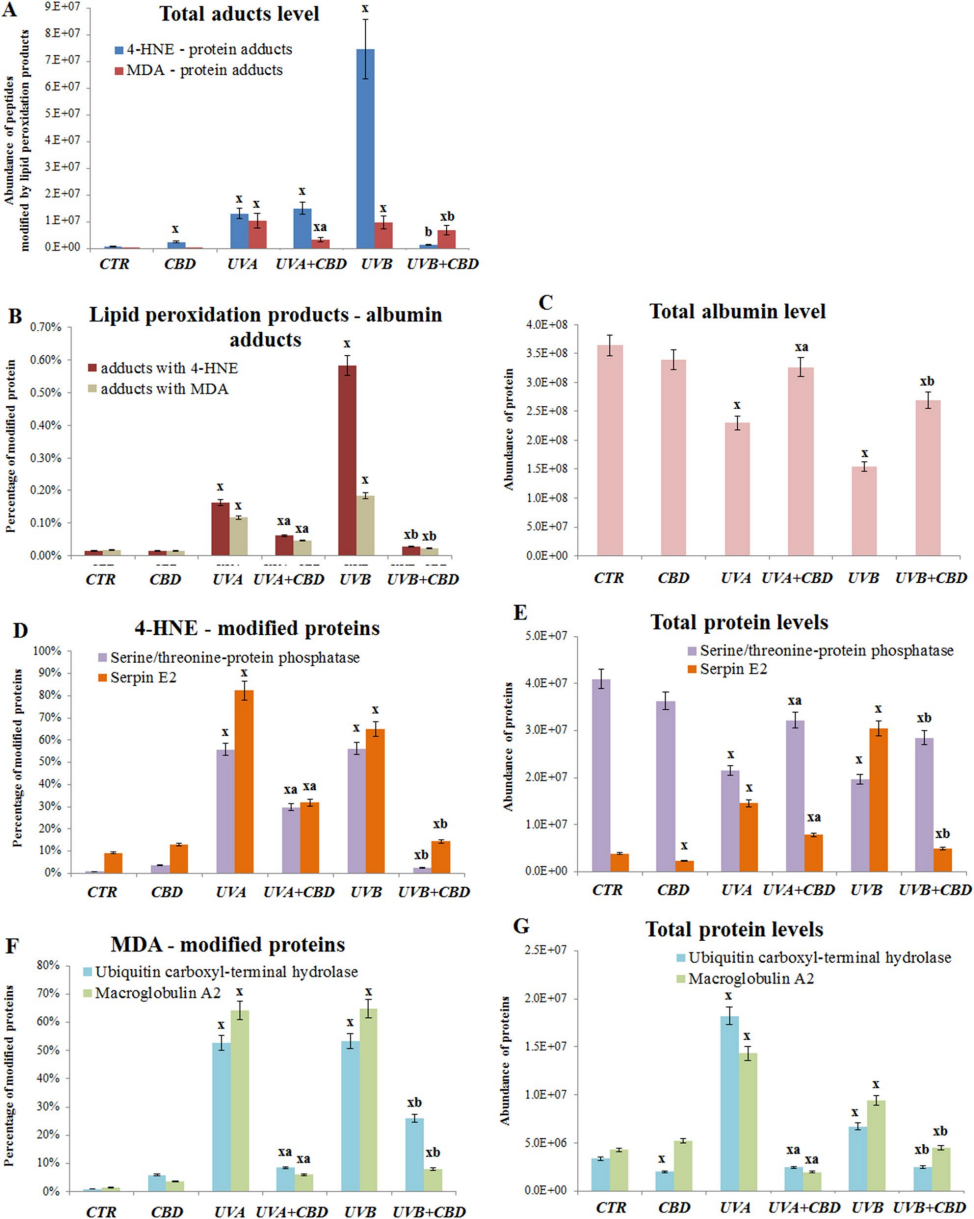
The level of lipid peroxidation products—proteins adducts in plasma from the control rats (CTR) and animals topically treated with cannabidiol (2.5 g CBD in 100 g petrolatum) and/or irradiated with UVA (increasing doses from 0.5 to 5 J/cm^2^) or UVB (increasing doses from 0.02 to 2 J/cm^2^). Individual diagrams show total level of adducts (**A**), level of albumin and its modifications (**B, C**), and levels of mostly modified proteins by 4-HNE (**D, E**) and MDA (**F, G**). Mean values ± SD of six independent experiments are presented. ^x^Statistically significant differences vs. CTR group, *p* < 0.05; ^a^Statistically significant differences versus UVA irradiated group, *p* < 0.05; ^b^Statistically significant differences versus UVB irradiated group, *p* < 0.05.

The effects of UVA and UVB rats skin chronic irradiation most likely caused in animals plasma the oxidative stress manifested by increased level of lipid peroxidation products—protein adducts formation. That was observed as 4-HNE or MDA—proteins adducts formation especially following UVA and UVB irradiation (Fig. 4A). However, CBD treatment was the most effective in decreasing 4-HNE—protein adducts level following UVB irradiation, while this effect was not observed in UVA irradiated group. On the other hand, in the case of MDA—protein adducts, CBD protective action was stronger following UVA irradiation (Fig. 4A). One of the most significantly modified by lipid peroxidation products proteins was albumin (Fig. 4B). UVA and UVB radiation led to the formation 4-HNE-albumin, as well as MDA-albumin adducts, however, the strongest effect was observed in the case of 4-HNE-albumin adducts in plasma of UVB irradiated rats. In addition, UV radiation itself lowers the level of this protein (Fig. 4C), however, in all cases CBD significantly prevented UV induced changes (Fig. 4B,C). Also other proteins were modified by lipid peroxidation products, including: serine/threonine-protein phosphatase and serpin E2 or ubiquitin carboxyl-terminal hydrolase and macroglobulin A2, which were modified even in 80% of total amount (Fig. 4D,F). For all these proteins CBD treatment prevented these modifications, as well as influenced also total level of mentioned proteins (Fig. 4E,G).

**Figure 5:**
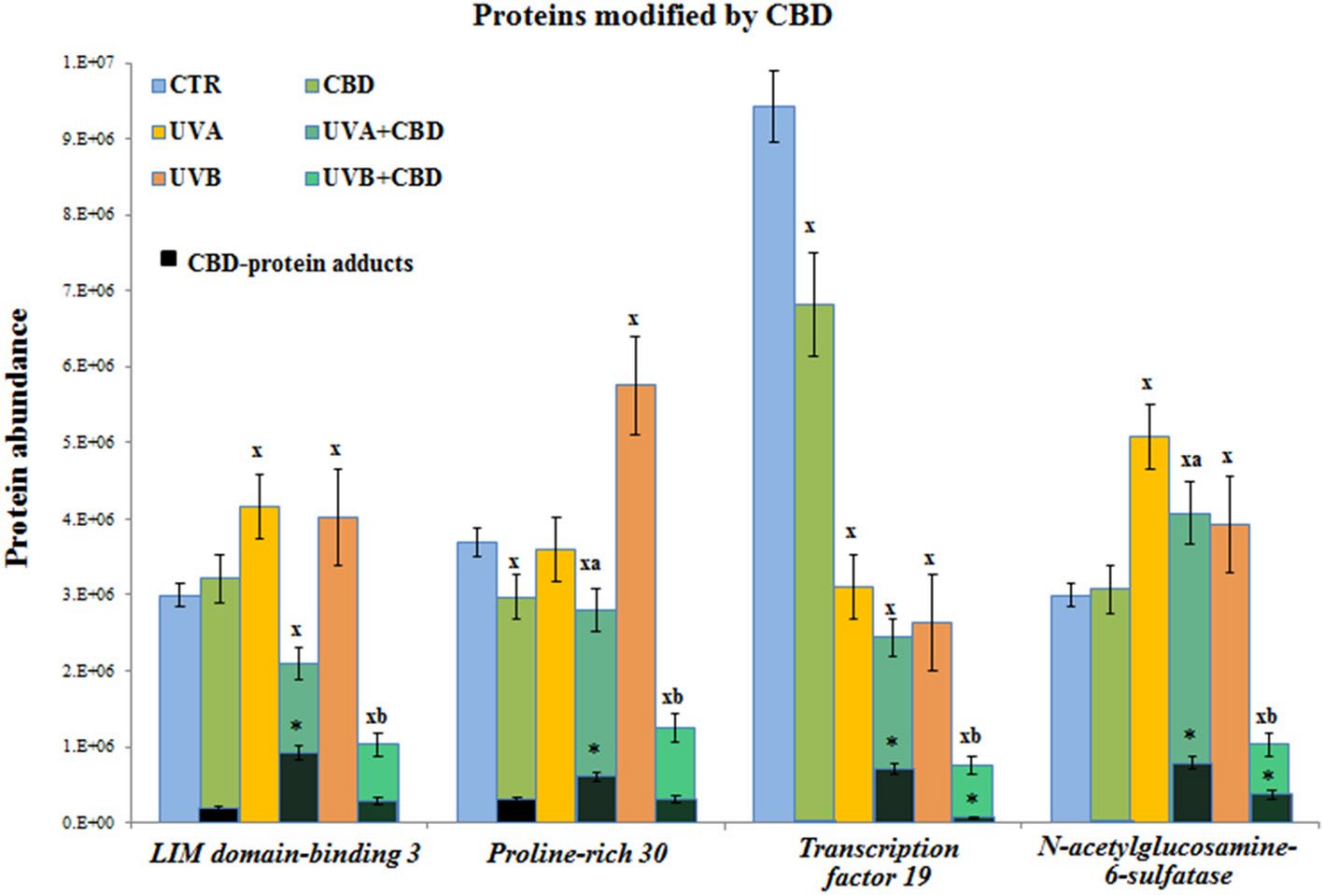
The level of proteins modified by cannabidiol (CBD) in plasma from the control rats (CTR) and animals topically treated with cannabidiol (2.5 g CBD in 100 g petrolatum) and/or irradiated with UVA (increasing doses from 0.5 to 5 J/cm^2^) or UVB (increasing doses from 0.02 to 2 J/cm^2^). Mean values ± SD of six independent experiments are presented. ^x^Statistically significant differences vs. CTR group, *p* < 0.05; ^a^Statistically significant differences vs. UVA irradiated group, *p* < 0.05; ^b^Statistically significant differences vs. UVB irradiated group, *p* < 0.05; *Statistically significant differences vs. CBD treated group, *p* < 0.05.

CBD skin topical treatment leading to the penetration of CBD into the blood also caused direct modifications to the plasma protein structure by creating CBD adducts (Fig. 5). The main proteins that bind CBD were LIM domain-binding 3, proline-rich protein 30, transcription factor 19, and N-acetylglucosamine-6-sulfatase. Moreover, the level on CBD-protein adducts was dependent on the UV radiation type that animals were exposed to. UVA radiation was conducive for CBD-protein adducts formation stronger than UVB, despite of the fact of different direction of action on the level of total expression of these proteins (Fig. 5).

## Discussion

The harmful impact of UV radiation on skin, as well as all body tissues, including disturbances in redox balance^12^ and blood pressure disorders^15^, push people to constantly look for ways to neutralize these effects. An easy and convenient way seems to be the topical skin application of CBD, that is more and more frequently used in a commercial oils/creams for skin care^40^. CBD has been also used in the treatment of most frequent skin disorders: psoriasis and atopic dermatitis, where it significantly improved both the skin parameters and the systemic symptoms^41^. On the other hand, many non-controlled use of CBD has been also reported in the case of human skin disease—epidermolysis^42^ or pain treatment^43^. Moreover, described self-induced CBD applications can affect in an indefinite way not only on cell metabolism but also the entire human body. The experiments on mouse and rat models showed that topically applied CBD penetrates in to the blood and is transported throughout the bloodstream^27,44^. Additionally, it has been shown in another animal experiment that spot application of CBD shows even greater effects comparing to its systemic distribution^45^.

The results of this study indicate appearance of keratin in the plasma of UVA/UVB irradiated animals. Keratin is a protein mainly synthesized by the epithelial cells. Because of that, it usually occurs intracellularly and its release into the blood occurs only upon skin or liver injury^46,47^. Therefore, keratin presence in plasma is considered even as a marker of the development of diseases such as normoglycaemia^48^ or Alzheimer’s disease^49^. In presented experiment UV-induced expression of keratin in plasma might resulting from skin cells damages^10^, as well as liver metabolism disruption^13^. However, despite of the source, this protein activates peripheral blood T cells, what induces systemic inflammation^50^. On the other hand, CBD topical treatment of UVB irradiated rats significantly prevents keratin release to the plasma. CBD protective effect direct on skin cells prevents their apoptosis following UVB irradiation^51^ and therefore fewer keratin can be released into the plasma. That is consistent with other reports on the anti-inflammatory effect of CBD^19^ and additionally indicates the new pathway of CBD action. Moreover, UVA and UVB radiation increases the expression of pro-inflammatory protein S100. This molecule exist in the form of the low stable heterodimer S100A8/A9, and both of its components can bind Ca^2+^ and during inflammation are released to the blood from neutrophils and monocytes to stimulate leukocytes for cytokine secretion^52^. Increased level of S100 in plasma so far has been believed to be accompanying the melanoma development process especially following skin exposure to the UVB radiation^53^, however, CBD partially prevents increase in S100 plasma level. Similar CBD action has been also observed in the case of in vitro UVB irradiated keratinocytes^54^, what significantly contributed to the reduction of the immune response of these skin cells. Simultaneously, UVA and UVB radiation decreased the expression of NFκB inhibitor—NFKBIL2 (IκB-related), a protein responsible for negative regulation of Toll-like receptors activated by NFκB and tumor necrosis factor α (TNFα) production prevention^55^. UVA, as well as UVB radiation by decreasing NFKBIL2 level in the plasma of experimental animals induces the pro-inflammatory signaling based on NFκB/TNFα pathway. However, anti-inflammatory action of CBD has been shown that is based on the NFκB as well as TNFα level decreasing^23,56^. The results of this study indicated that these changes might be dependent on Toll-like receptor inhibition by NFKBIL2, which level is enhanced by CBD in plasma of both not irradiated and UV treated animals. Similar action of CBD has been previously observed in the case of lipopolysaccharide-stressed cells, where CBD concentration dependent increase in other NFκB inhibitor—IκB occurred^57^.

Rats exposure to the UV radiation causes changes throughout the organism, not only in the context of a pro-inflammatory reaction, but also other biologically important processes. That starts at the signaling level, where UV radiation significantly decreases the expression of many intercellular factors, including 14-3-3 protein and protein kinase C (PKC). 14-3-3 protein has ability to binding other proteins such as kinases, phosphatases, or transmembrane receptors thus additionally increasing the scope of its activities^58^. On the other hand, 14-3-3 protein inhibits cell cycle progression by arrests cell at G1/S- and G2/M-transition^59^, therefore its decreased level in plasma by UV radiation is additionally dangerous due to the risk of developing carcinogenic signaling. Also UV decreased PKC expression regulates numerous cellular responses including gene expression, protein secretion, cell proliferation, as well as cell cycle progression. PKC, similarly to 14-3-3 can stopped cells at G1/S- and G2/M-transition, and again the decrease level of this protein in plasma carries the risk of neoplastic processes^60^. Similar results were observed in UV irradiated fibroblasts^61^, however, opposite data from UV irradiated keratinocytes, showed that radiation activates PKC for the apoptosis induction in the damaged skin cells^62^. CBD treatment significantly prevents mentioned changes in the cases of this both mentioned molecules: 14-3-3 protein and PKC, as well as other presented proteins that expressions are decreased by UV radiation. Moreover, CBD can not only enhance the expression of PKC but also activates this enzyme by the direct interaction with serotonin receptor (5-HT_1A_R)^63^. Moreover, literature data indicate that cannabinoids significantly arrest cells in G2/M-transition^64^, which does not rule out a potential role of the 14-3-3 protein or PKC in this regulation.

Chronic UV skin irradiation is such a highly harmful environmental factor that its negative effect is also reflected in the redox status of the whole organism, including the blood. Oxidative stress in the blood leads to increased lipid peroxidation and, consequently to lipid peroxidation products-protein adducts formation, that are most intensive in the case of albumin^65^. Human albumin is a protein that plays an important role in the physiological transport of many compounds such as free fatty acids, steroids, and some metals and drug metabolites. This protein contains numerous of nucleophilic residues that react with high reactive electrophilic aldehydes, including 4-HNE and MDA^65,66^. 4-HNE- as well as MDA-albumin adducts has been found in the plasma of patients with? Various diseases, including diabetes, atherosclerosis, or liver diseases^67–69^. In this study, plasma 4-HNE- and MDA–albumin adducts, for the first time, are described as a results of direct skin UV irradiation. Interestingly, less penetrating UVB radiation causes the stronger increase in the formation of lipid peroxidation products-albumin than UVA. This relationship concerns adducts between 4-HNE and albumin. Moreover, albumin is an attractive target for biomarker studies of oxidative stress because it is highly abundant, constituting over half the total serum protein^70^. As a positive effect of albumin modifications other plasma proteins can be protected from oxidation^71^, also induced by UV skin irradiation. Obtained data presents that CBD partially prevents 4-HNE and MDA—albumin adducts formation. CBD as an antioxidant molecule can both prevent the generation of reactive electrophiles^19^, as well as it can reduce the possibility of their reaction with other proteins to form adducts^54^. Due to that, CBD counteract UV-induced changes in structure of plasma proteins. That concerns not only on albumin, but also on other modified proteins, like serine/threonine-protein phosphatase, serpin E2, ubiquitin carboxyl-terminal hydrolase, and macroglobulin A2, that are founded in this study as the main molecules modified by lipid peroxidation products following UV radiation. As it has been described before this type of protein modifications induce various effects on the biologically important molecules, what often lead to the pro-inflammatory or pro-apoptotic signals generation^72^. Therefore, the main molecules involved in signal transduction are most exposed to interaction with lipid peroxidation products. That include also proteins from the phosphorylation and dephosphorylation (kinase-phosphatase) system, therein serine/threonine-protein phosphatase, responsible for removal of phosphate groups and regulation cell proliferation, cell differentiation and apoptosis^73^. So far it has been described that UV radiation and oxidative stress can directly induce phosphatases activity^74,75^. Moreover, increased level of 4-HNE-modified proteins reduces oxidation of phosphatases and stimulates their activity, in response to the oxidative stress^76^. Other studies also show that the phosphorylation and dephosphorylation system in plasma under stress conditions can be influenced by lipid peroxidation products—protein adduct formation by 4-HNE binding to the kinases^36^, what differently affects the activity of these enzymes. Generally, the formation of 4-HNE-kinase adducts only at non-toxic concentrations activate phosphorylation usually triggering the pro-protective signal transmission cascade^77^. In the case of high 4-HNE-protein adducts concentrations reverse action is expected, what in conjunction with activation phosphatases activation will likely block this signaling pathway. This is particularly important due to the fact that CBD is potent in reducing the level of serine/threonine-protein phosphatase modification, particularly following high-energetic and harmful UVB radiation.

Other plasma protein significantly modified by 4-HNE following animal irradiation is serpin E2, an important protease inhibitor, that take a part in inflammation and coagulation regulation. The level of serpins is up-regulated following UV irradiation highlighting it essential role in the pro-apoptotic signal transduction^78^. Other study shows that also CBD can enhance serpins expression in macrophages and lung cancer cells^79^. The biological activity of serpin depends on its conformation, and can be lost due to the modifications of some exposed nucleophilic lysine residues (Lys-11, Lys-114, Lys-125), which were previously found as a target for the attachment of acrolein—another electrophilic aldehyde generated during lipid peroxidation^80^. Therefore, the lowering level of 4-HNE-serpin E2 adducts by CBD, may indicate its protective role against disturbances in the body's natural defense mechanisms against oxidative stress based on serpins activity. However, the exact mechanism of action remains unclear. Additionally, the coagulation process in rats irradiated with UV is disturbed by the increased level of macroglobulin A2 (A2M), which is an important anti-proteolytic inhibitor of coagulation^81^. Surprisingly, UV-induced MDA binding to A2M may attenuate the proteinases action^82^ and favor coagulation processes. CBD significantly reduces both the level of A2M as well as the percent of modified molecules. Other results show that changes in plasma accompanying pathologic conditions, e.g. rheumatoid arthritis, also are characterised by decreased level of MDA-A2M adducts, suggesting the organism's natural protective reaction against oxidative stress and inflammatory processes^83^, that in UV irradiated rats is supported by CBD.

UV radiation leading to the oxidative modifications of proteins, changes their structure so strongly that the process of their degradation is disturbed. That favors the accumulation of dysfunctional molecules, which hinder the normal functioning of the whole organism^84^. Moreover, UV radiation increases the expression of ubiquitin carboxyl-terminal hydrolase, that, due to its deubiquitinating properties additionally prevents proteasomal digestion of damaged proteins marked for degradation. However, oxidative modifications, which includes MDA–protein adducts formation, have been found as a factors that inhibit deubiquitination processes^85^. In animals treated with CBD following UVA irradiation a significant decrease in MDA-ubiquitin carboxyl-terminal hydrolase adducts level is observed, what should be accompanied with high hydrolase activity and lowering the level of ubiquitination, resulting from CBD protection against proteins modification under stress conditions.

Obtained in this study results indicate also the CBD–protein adducts formation with plasma molecules. So far, little work has focused on the direct effects of CBD on the structure and activity of the proteins, however, it has been found to interact with various pathways. Recent publications show, among others, that CBD has significant effect on the cellular antioxidant system: by adducts creation with Keap1, stimulates antioxidant and cytoprotective transcription factor Nrf2^23^ or, on the other hand, by direct binding with GSH or cytochrome P450, CBD inactivate the antioxidant properties of these molecules^38^. On the other hand, CBD-kinase adducts formation significantly stimulates the activity of AKT and PI3K in mice spinal cord cells^86^ or human ligament stem cells^87^. Obtained results indicate that CBD can additionally create adducts with LIM domain-binding 3, cytosolic protein responsible for cytoskeleton organization. However, this protein found in plasma suggests significant cells damage and favors cells adhesion and localization changing, what explains the increase in this protein level in UV irradiated animals. Moreover, LIM domain-binding 3 is a protein that in active form binds kinase C and A^88^, which, as mention before, similarly to LIM domain-binding 3 also can create adducts with CBD. Therefore, the cytoprotective action of CBD following UV irradiation is observed as a decreased level of LIM domain-binding 3 from damaged cells, as well as high level of adducts with this protein, probably stimulating its activity in cells adhesion.

Another cellular protein, modified by CBD in rats plasma is proline-rich protein 30. It is a molecule strongly connected with signal transduction and cytoskeletal proteins, including its actin‐binding properties, kinase PI3K or calcium channel regulation^89^. Proline‐rich sequences are commonly found in situations requiring the rapid recruitment or interchange of several proteins, such as during initiation of transcription, signaling cascades, and cytoskeletal rearrangements^89^. However, in the case of this protein, CBD by adducts formation blocks its domains, leading to its inactivation, thus reducing the importance of this protein in the plasma of animals exposed to UV radiation. On the other hand, CBD also attaches to N-acetylglucosamine-6-sulfatase, a lysosomal enzyme involved in the glycosaminoglycans catabolism in the body. It has been found that N-acetylglucosamine-6-sulfatase activity is involved in the inhibition of heparin-based anti-coagulation^90^. On the other hand, it is know that UV radiation can induce a coagulation^91,92^. Unfortunately, there is currently no information on how the formation of adducts with CBD affects the activity of this enzyme, however, other studies show that cannabinoids can increase the activity of other sulfatases^93^. Modified by CBD is also transcription factor 19, a potential trans-activating factor that could play an important role in the transcription of genes required for the later stages of cell cycle progression^94^. However, its activity effects and structure modifications, especially in plasma, are not well described, so it cannot be suggested how CBD adducts creation influences its action.

In conclusion, the results of this in vivo study confirm the in vitro results to date, indicating the complementary anti-inflammatory and antioxidant effects of CBD. Presented results indicate that the mechanism of CBD biological action in vivo includes not only preventing skin cell damage, but also its direct effect on plasma proteins, resulting in the inhibitory effect of CBD on pro-inflammatory signaling throughout the body. In addition, this phytocannabinoid is effective in preventing oxidative stress and oxidative modification of the protein profile and structure. Consequently, CBD may be a compound used to protect the organism against the harmful effects of UV radiation. However, its operation requires careful analysis to ensure that it does not pose a metabolic hazard to other cells or tissues of the animal or human.

## Supplementary Information

Supplementary Information 1.

Supplementary Information 2.

Supplementary Information 3.

## Data Availability

The MS proteomics data are available via ProteomeXchange with identifier PXD026668.

## Abbreviations

4-HNE: 4-Hydroxynonenal
A2M: Macroglobulin A2
AKT: Protein kinase B
ANOVA: Analysis of variance
ARHGAP18: GTPase-activating protein 18
CBD: Cannabidiol
CTR: Control
EDTA: Ethylenediaminetetraacetic acid
ESI: Electrospray ionization source
GSH: Glutathione
GSSG: Glutathione disulfide
ILK: Integrin-linked protein kinase
IκB: Inhibitor of nuclear factor κB
LC–MS/MS: Liquid chromatography–tandem mass spectrometry
Lys: Lysine
MDA: Malondialdehyde
NFKBIL2: Nuclear factor of κB-like 2
NFκB: Nuclear factor κB
PA2G4: Proliferation-associated protein 2G4
PCA: Principal component analysis
PGAM2: Phosphoglycerate mutase 2
PI3K: Phosphoinositide 3-kinase
PKC: Protein kinase C
RBP: Retinol-binding protein
SDS-PAGE: Sodium dodecyl sulfate polyacrylamide gel electrophoresis
STIP1: Stress-induced-phosphoprotein 1
TNFα: Tumor necrosis factor α
UV: Ultraviolet

## Code availability

2rwnmK3m.
