# Supplementary material for: UV induced changes in proteome of rats plasma are reversed by dermally applied cannabidiol
Source: Sci Rep. 2021 Oct 19;11:20666. doi: 10.1038/s41598-021-00134-8 (PMC8526570; doi:10.1038/s41598-021-00134-8)
Supplement: Supplementary file 2 — Supplementary Information 2. [file 41598_2021_134_MOESM2_ESM.docx]

**Supplementary figure S1.**

The spectrum of UV emitters (PL-S 9W; Philips, Amsterdam, Netherlands) used in experiment for skin on rat’s back irradiation. Data officially provided by the manufacturer.

1. UVA (max. emission peak at 368 nm)

1. UVB (max. emission peak at 311 nm)
