# Supplementary material for: UV induced changes in proteome of rats plasma are reversed by dermally applied cannabidiol
Source: Sci Rep. 2021 Oct 19;11:20666. doi: 10.1038/s41598-021-00134-8 (PMC8526570; doi:10.1038/s41598-021-00134-8)

**Supplementary figure S2**.

SDS-PAGE separation and staining with Coomassie Brilliant Blue R-250 of plasma proteins from the control rats (CTR) and animals topically treated with cannabidiol (2.5g CBD in 100g petrolatum) and/or irradiated with UVA (increasing doses from 0.5 to 5 J/cm^2^) or UVB (increasing doses from 0.02 to 2 J/cm^2^). Full-length image of the gel.


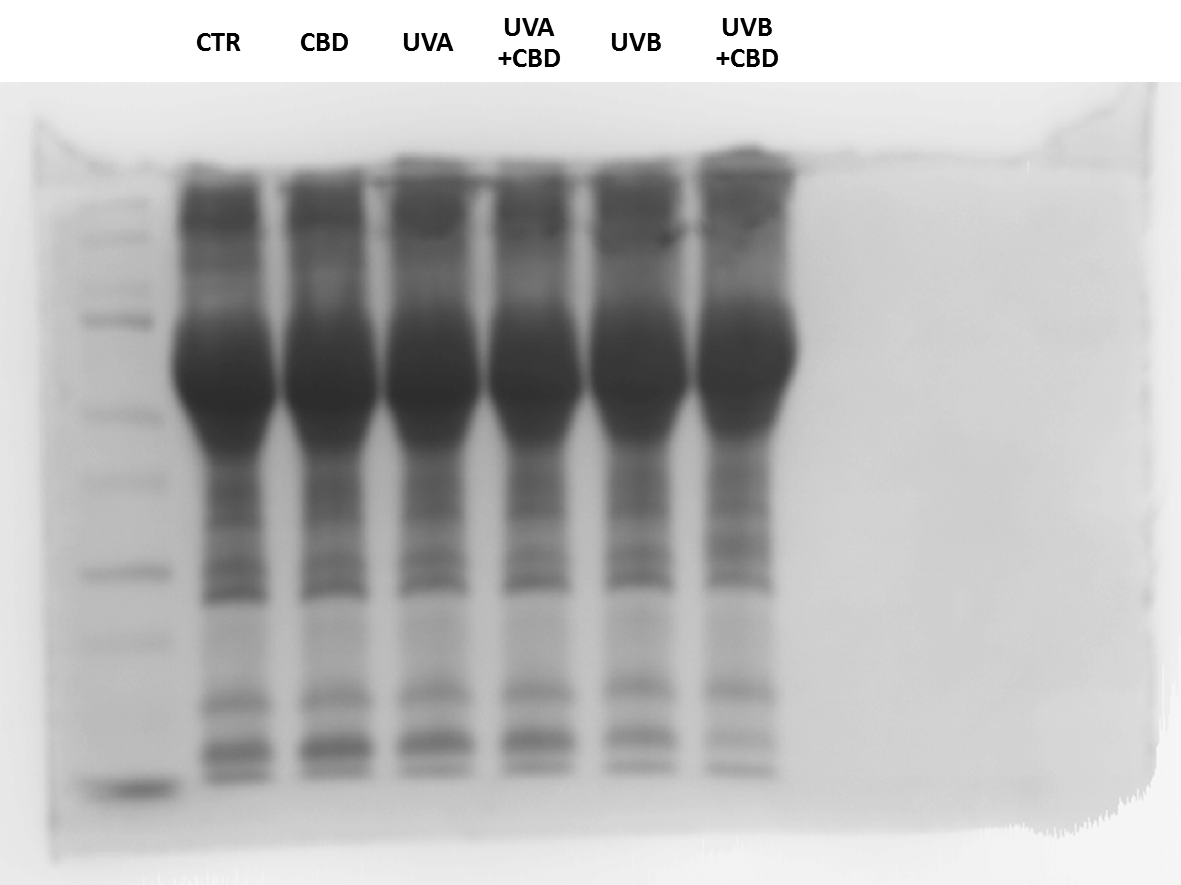

Supplement: Supplementary file 3 — Supplementary Information 3. [file 41598_2021_134_MOESM3_ESM.docx]
